# A retrospective study of deep learning generalization across two centers and multiple models of X-ray devices using COVID-19 chest-X rays

**DOI:** 10.1038/s41598-024-64941-5

**Published:** 2024-06-25

**Authors:** Pablo Menéndez Fernández-Miranda, Enrique Marqués Fraguela, Marta Álvarez de Linera-Alperi, Miriam Cobo, Amaia Pérez del Barrio, David Rodríguez González, José A. Vega, Lara Lloret Iglesias

**Affiliations:** 1 Departamento de Radiología, Hospital Universitario Rey Juan Carlos, Calle Gladiolo, s/n, 28933 Móstoles, Spain; 2 Departamento de Tecnologías de La Información, Universidad CEU San Pablo, Calle Julián Romea, 22, 28003 Madrid, Spain; 3 Departamento de Radiofísica y Protección Radiológica, Hospital Universitario Marqués de Valdecilla, Avenida de Valdecilla s/n, Santander, Spain; 4 Departamento de Otorrinolaringología, Clínica Universidad de Navarra, Calle del Marquesado de Santa Marta, 1, 28027 Madrid, Spain; 5 Advanced Computing and E-Science Research Group, Institute of Physics of Cantabria, Grupo de Computación y E-Ciencia, CSIC-UC, IFCA-CSIC, Avenida de los Castros s/n, 39005 Santander, Spain; 6 Servicio de Radiología, Complejo Hospitalario de Navarra, C. de Irunlarrea, 3, 31008 Pamplona, Spain; 7 Departamento de Morfología y Biología Celular, Grupo SINPOS, Universidad de Oviedo, Avenida Julián Clavería, 6, 33006 Oviedo, Spain; 8 Facultad de Ciencias de La Salud, Universidad Autónoma de Chile, Avenida Pedro de Valdivía, 425, 751 1185 Providencia-Santiago de Chile, Chile

**Keywords:** Computer science, Radiography

## Abstract

Generalization of deep learning (DL) algorithms is critical for the secure implementation of computer-aided diagnosis systems in clinical practice. However, broad generalization remains to be a challenge in machine learning. This research aims to identify and study potential factors that can affect the internal validation and generalization of DL networks, namely the institution where the images come from, the image processing applied by the X-ray device, and the type of response function of the X-ray device. For these purposes, a pre-trained convolutional neural network (CNN) (VGG16) was trained three times for classifying COVID-19 and control chest radiographs with the same hyperparameters, but using different combinations of data acquired in two institutions by three different X-ray device manufacturers. Regarding internal validation, the addition of images from an external institution to the training set did not modify the algorithm’s internal performance, however, the inclusion of images acquired by a device from a different manufacturer decreased the performance up to 8% (*p* < 0.05). In contrast, generalization across institutions and X-ray devices with the same type of response function was achieved. Nonetheless, generalization was not observed across devices with different types of response function. This factor was the key impediment to achieving broad generalization in our research, followed by the device’s image-processing and the inter-institutional differences, which both reduced generalization performance to 18.9% (*p* < 0.05), and 9.8% (*p* < 0.05), respectively. Finally, clustering analysis with features extracted by the CNN was performed, revealing a substantial dependence of feature values extracted by the pre-trained CNN on the X-ray device which acquired the images.

## Introduction

Chest radiography is a widely used imaging modality in the regular medical practice for the evaluation, management and follow-up of patients with several diseases, such as COVID-19 pneumonia^1^. However, chest radiography is a complex imaging modality to interpret^2^, and its evaluation requires experience and expertise^3^. Deep learning (DL) algorithms have potential to improve the quality of radiographic interpretation and lead to more accurate diagnoses^3^.

Several DL algorithms for chest radiographs (CXRs) classification have been published in the past years, especially in the context of COVID-19 pandemic^4–6^. However, these algorithms often show low generalization performance when data distribution shifts^7–12^.

This generalization deficiency is often overlooked during algorithm evaluation, because algorithms are often assessed on test subsets that come from the same population sample as the training and validation subsets. Therefore, typically only the internal validation performance is estimated, and generalizability is not usually properly evaluated. For this reason, DL algorithms’ performance should also be assessed on test subsets coming from a different source from which training and validation subsets were obtained^7–12^.

In medical data, the difference between the source from which training, validation, and usually test subsets are obtained and the real-world environment can cause particularly considerable distribution shifts, which may lead DL networks to significantly reduce their generalization performance^9,10,12^. This issue remains a major challenge in machine learning research^13^.

Nevertheless, generalization deficiency for image classification in DL networks has only been discussed by a few authors in the medical field. While the causes remain unclear, most of these authors have only studied generalization through testing different DL networks on external datasets from external institutions^8,10,12,14^. Unlike previous works, this novel research separately assesses the influence of institution and X-ray device on both algorithm’s internal validation and generalization performances.

For this purpose, factors that may potentially affect the performance of DL networks were divided into two categories:

- X-ray device related factors: aspects that affect image pixel values, which include the acquisition protocol, the type of response function of the radiology device, and the image processing applied by the X-ray device.
- Institutional related factors: differences among hospitals that do not change the pixel values, such as labeling criteria, population demographics, disease epidemiology, and radiology workflow.

Thus, this work is aimed to study the role of the aforementioned factors, but is not intended to provide a new DL algorithm for CXR classification, as there are already plenty of these in the literature which show satisfactory performance, particularly, in internal validation. In contrast, this novel study aims to evaluate the influence of X-ray device associated and institutional related factors on DL algorithms performance both in internal validation and generalization.

To achieve this purpose, one DL network will be trained and tested using different subsets in which the variables institution and X-ray device, (including device’s image processing and response function) will be completely controlled. This represents a differentiating issue from other previous publications, as to the best of our knowledge, it is the first time that these variables are fully controlled.

## Materials and methods

**Figure 1:**
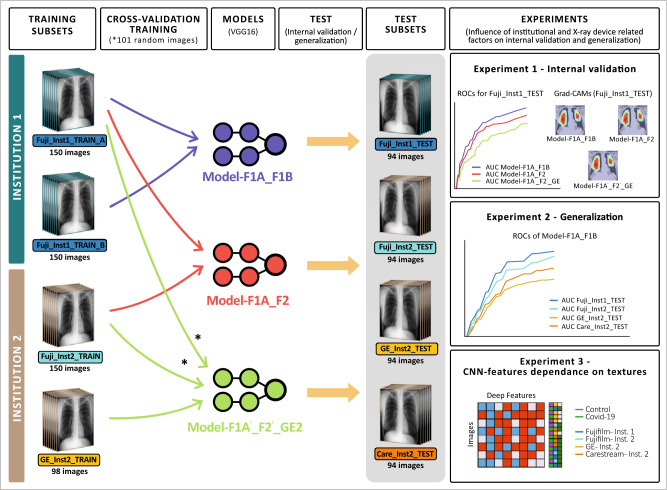
Experimental design.

Three experiments were carried out to study the influence of institutional and X-ray device related factors on the internal validation and generalization performance of a DL network for CXR classification (Fig. 1).

### Ethical approval

This research involved patients from two different medical institutions: Hospital Universitario Marqués de Valdecilla, located in Santander, Cantabria, Spain—referred to as Institution 1 in the text; and Hospital de Sierrallana in Torrelavega, Cantabria, Spain—referred to as Institution 2. The Ethics Committee of both institutions, Comité de Ética de la Investigación con Medicamentos y Productos Sanitarios de Cantabria, approved this research. Since this study was approved by the Comité de Ética de la Investigación con Medicamentos y Productos Sanitarios de Cantabria, without direct interaction with patients or use of tissue samples, and using retrospective images acquired in the past and anonymized, informed consent was not required^15^. All methods reported in this work were carried out in accordance with the pertinent guidelines and regulations.

### Dataset and subsets

Images for this research were all frontal view CXRs manually labeled by three expert radiologists, with more than 5 years of experience, in two classes (COVID-19 and Control), according to the inclusion criteria summarized in Table 1. In the text, these classes are named as target classes.

**Table 1:** Inclusion criteria for target classes.

Target class	Medical history	Imaging findings	Follow-up
COVID-19	Positive PCR testing	Three radiologists, where at least one was a thoracic radiologist, reported COVID-19 findings on the CXR	Progression of imaging opacities were verified in a follow-up CXR
Control	No COVID-19 symptoms	Three radiologists, with at least one thoracic radiologist, reported no pathological findings on the CXR	

Meeting all the inclusion criteria was required for an image to be included in a class.

*PCR* polymerase chain reaction, *CXR* chest radiograph.

The main image dataset was created by simple random sampling from four image databases, as described in Supplementary Appendix A1. This main dataset contained images acquired by three different X-ray devices in two institutions: 394 images acquired by a Fujifilm FDR smart FGX device in institution 1; 244 images acquired by the same device model (Fujifilm FDR smart FGX) in institution 2; 192 images acquired by a general electric (GE) revolution XRD device in institution 2; and 94 images acquired by a Carestream DRX Evolution Plus device in institution 2. Note that Fujifilm and Carestream devices have the same type of response function (logarithmic), while GE has a different type (linear)^16,17^.

Finally, eight subsets described in Table 2 were created by random sampling without repetition of the main dataset. Random sampling was performed with stratification to ensure an equal distribution of COVID-19 and Control images within each subset (50 percent of each class).

**Table 2:** Image subsets.

Subset	X-ray device model	Institution	Number of images
Train subsets			
Fuji_Inst1_TRAIN_A	Fujifilm FDR Smart FGX	Institution 1	150
Fuji_Inst1_TRAIN_B	Fujifilm FDR Smart FGX	Institution 1	150
Fuji_Inst2_TRAIN	Fujifilm FDR Smart FGX	Institution 2	150
GE_Inst2_TRAIN	General Electric Revolution XRD	Institution 2	98
Test subsets			
Fuji_Inst1_TEST	Fujifilm FDR Smart FGX	Institution 1	94
Fuji_Inst2_TEST	Fujifilm FDR Smart FGX	Institution 2	94
GE_Inst2_TEST	General Electric Revolution XRD	Institution 2	94
Care_Inst2_TEST	Carestream DRX Evolution Plus	Institution 2	94

Note that Fujifilm and Carestream devices have a different image processing, but the same type of response function (logarithmic). By contrast, general electric device has a different image processing and a different type of response function (linear).

### Image preprocessing

Images were collected as 16-bit unsigned integer monochrome pixels stored in DICOM format. After data collection, images were resized to 512 × 512 pixels using cubic spline interpolation, and pixel values were rescaled to [0, 1].

### Experiments to test the influence of institutional and device related factors

#### Experiment 1: evaluation of internal validation performance

The first experiment analyzed the influence of institutional and device related factors on the internal validation performance of a DL algorithm (Fig. 1). For this purpose, the same DL network (a VGG16) was trained three times for the classification of CXR images. Each time, with the same architecture, hyperparameters (details in Supplementary Appendix A2), and number of images (300), but using different training subsets (Table 3).

**Table 3:** Trainings and models.

Training	Model	Training set	Total number of training images
1	Model-F1A_F1B	Fuji_Inst1_TRAIN_A + Fuji_Inst1_TRAIN_B	300
2	Model-F1A_F2	Fuji_Inst1_TRAIN_A + Fuji_Inst2_TRAIN	300
3	Model-F1A’_ F2’_GE2	Fuji_Inst1_TRAIN_A_101* + Fuji_Inst2_TRAIN_101** + GE_Inst2_TRAIN	300

Models were all trained with the same architecture (VGG16) and hyperparameters, and they were named with a reference of the subsets used (F1A = Fuji_Inst1_TRAIN_A; F1A’ = 101 random images from Fuji_Inst1_TRAIN_A, etc.)

*101 random images from Fuji_Inst1_TRAIN_A.

**101 random images from Fuji_Inst2_TRAIN.

First training was performed with subsets Fuji_Inst1_TRAIN_A and Fuji_Inst1_TRAIN_B, so it includes 300 images acquired by a Fujifilm FDR smart FGX device from institution 1. The resulting model was named Model-F1A_F1B, because of the subsets used (F1A = Fuji_Inst1_TRAIN_A, etc.).

Second training included subsets Fuji_Inst1_TRAIN_A and Fuji_Inst2_TRAIN, so it was performed with all images acquired by a Fujifilm FDR smart FGX device, but half in institution 1 and half in institution 2. This model was named Model-F1A_F2.

The third and last training was performed with 101 random images from Fuji_Inst1_TRAIN_A, 101 random images from Fuji_Inst2_TRAIN, and the 98 images from GE_Inst2_TRAIN. This means that the resulting model, named Model-F1A’_ F2’_GE2, was trained including images acquired by two different manufacturers, Fujifilm FDR smart FGX and general electric (GE) revolution XRD, and in two different institutions (1 and 2).

Later, internal validation performance of these three models were tested on Fuji_Inst1_TEST subset, which contained all images acquired by a Fujifilm FDR smart FGX device in institution 1. This subset was the only test subset that could give results of internal validation for the three models, since all models were trained using sets of images which contained CXRs acquired by a Fujifilm FDR smart FGX device in institution 1. Finally, the internal validation performances of the three models were compared to assess the influence of institutional and X-ray machine related factors.

The influence of institutional factors was studied by comparing performances of Model-F1A_F1B and Model-F1A_F2, as both of them were trained with 300 images of the same X-ray device model, but Model-F1A_F1B coming from institution 1, and Model-F1A_F2 coming from institutions 1 and 2. Therefore, performance differences among these models could be probably attributable to institutional related factors.

The influence of X-ray machine related factors was assessed comparing the performances of Model-F1A’_ F2’_GE2 with Model-F1A_F1B and Model-F1A_F2. While Model-F1A_F1B and Model-F1A_F2 were trained with 300 images acquired by a Fujifilm FDR smart FGX, Model-F1A’_ F2’_GE2 was trained with 300 images, some of them acquired by a Fujifilm FDR smart FGX, and the rest by a GE revolution XRD device. Thus, this comparison revealed the effect of adding images from a different manufacturer to the training sample.

The metric used to evaluate the performances was the area under the receiver operating characteristic curve (AUC)^18^. Additionally, gradient-weighted class activation mapping (Grad-CAM) heatmaps were used to identify the significant regions in the image for making the prediction^19^. This analysis evaluated how the addition of images acquired in a distinct institution and by a different X-ray device affected the DL network’s ability to learn causal relationships.

#### Experiment 2: evaluation of generalization

The second experiment studied the influence of both institutional and device-related factors on a DL network’s generalization (Fig. 1). Model-F1A_F1B was evaluated on the four test subsets that included images from two different institutions and three distinct X-ray devices (Table 2). Therefore, four AUCs were obtained: AUC on subset Fuji_Inst1_TEST (internal validation); AUC on Fuji_Inst2_TEST (generalization to an external institution with the same X-ray device); AUC on Care_Inst2_TEST (generalization to an external institution with a X-ray device which has a different image processing but the same type of response function); and AUC on GE_Inst2_TEST (generalization to an external institution with a X-ray device which has a different image processing and different type of response function).

Later, considering the shifting factor, AUCs were compared to evaluate the influence of institutional factors on generalization (AUC-Fuji_Inst1_TEST vs AUC-Fuji_Inst2_TEST), as well as X-ray device factors, including device’s image processing (AUC-Fuji_Inst2_TEST vs AUC-Care_Inst2_TEST) and device’s type of response function (AUC-Care_Inst2_TEST vs AUC-GE_Inst2_TEST).

#### Experiment 3: evaluation of the dependence of CNN-features on textures

The third experiment investigated the influence of institutional and device related factors on the values of the features extracted by the pre-trained CNN (Fig. 1). The hypothesis was that radiological image textures depend on the X-ray device used to acquire the image. Differences in device´s image processing and response functions result in differences among imaging textures based on the X-ray device.

Therefore, feature values extracted by CNNs could also be highly dependent on the X-ray device that acquires the image. This issue could hinder the generalization of DL networks, making them only suitable to images obtained from the same devices as the ones used in training.

In this context, Model-F1A_F1B was used to extract features from the test subsets. Later, features from the last convolutional layer were clustered using a hierarchical clustering algorithm^20^, which was implemented using python’s scientific graphics library, seaborn (version 0.11.1)^21^. This unsupervised approach allowed us to examine which image classes were more evident for the CNN, namely target classes (COVID-19 and control) or other hidden classes (such as the X-ray device which acquired the image, or the institution where the images were obtained). To ensure that results were not biased by CXRs metallic tokens, this experiment was repeated by cropping the images to preserve only their central part.

This experiment aimed to evaluate whether the difference in pixel values between a COVID-19 image from a Fujifilm device and a COVID-19 image from a GE device is greater or smaller than the difference between a COVID-19 image and a control image, both acquired by the same X-ray device.

### Statistical analysis

Cross-Validated AUCs 95% confidence intervals (CIs) were computed with the R package cvAUC^22^. The AUC differences with their 95% CIs were calculated using the bootstrap method^23^. Any difference where the CI excluded the 0 value was considered to be statistically significant with a *p*-value < 0.05.

## Results

### Patients

This research included 874 patients, 45.1% (394) from institution 1 and the remaining from institution 2. The study sample comprised 42.6% (372) females and 57.4% (502) males. The median age was 62 years, while the average age was 60 ± 17 years (5–96). Detailed population descriptive statistics are summarized in Table 4.

**Table 4:** Descriptive statistics of population age and gender.

Subset	Age (years)					Gender % (n)		N
	Median	Mean	SD	Min	Max	Female	Male	
Fujifilm-Institution 1	59	58	17	16	93	44.2 (174)	55.8 (220)	394
Fuji_Inst1_ TRAIN _A	60	59	17	16	93	40.7 (61)	59.3 (89)	150
Fuji_Inst1_ TRAIN _B	57	56	17	17	89	54.0 (81)	46.0 (69)	150
Fuji_Inst1_TEST	61	60	16	24	92	34.0 (32)	66.0 (62)	94
Fujifilm-Institution 2	66	63	17	5	96	38.5 (94)	61.5 (150)	244
Fuji_Inst2_TRAIN	61	62	16	20	92	38.0 (57)	62.0 (93)	150
Fuji_Inst2_TEST	68	66	17	5	96	39.4 (37)	60.6 (57)	94
GE-Institution 2	63	60	18	18	91	43.8 (84)	56.3 (108)	192
GE_Inst2_TRAIN	63	60	18	18	87	42.9 (42)	57.1 (56)	98
GE_Inst2_TEST	63	60	18	19	91	44.7 (42)	55.3 (52)	94
Carestream-Institution 2	65	63	17	20	86	45.5 (20)	54.6 (24)	94
Care_InstT2_TEST	65	63	17	20	86	45.5 (20)	54.6 (24)	94
Total	62	60	17	5	96	42.7 (372)	57.4 (502)	924

*SD* standard deviation, *GE* general electric.

### Experiments to test the influence of institutional and device related factors

#### Experiment 1: evaluation of internal validation performance

**Figure 2:**
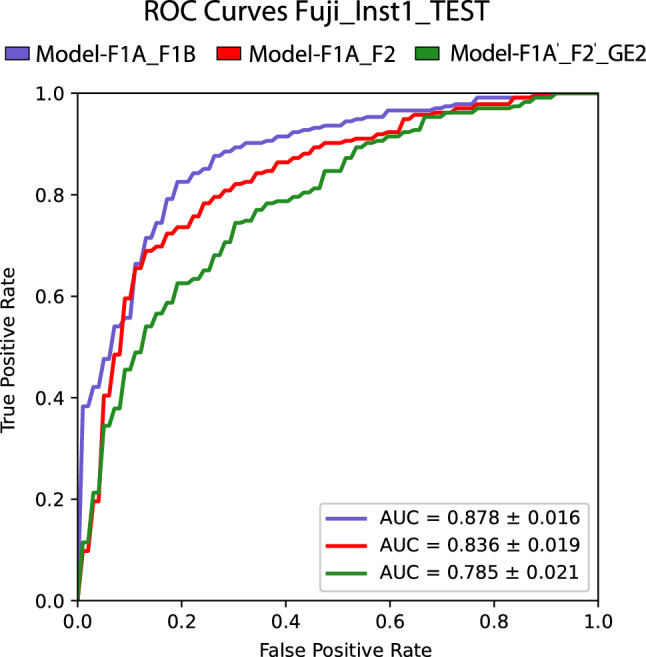
Receiver operating characteristic (ROC) curves on subset Fuji_Inst1_TEST (internal validation).

Internal validation performances of Model-F1A_F1B and Model-F1A_F2 did not show significant statistical differences (Fig. 2 and Table 5). Thus, the addition of images to the training sample acquired in a different institution by the same X-ray model did not have a significant impact on the algorithm’s internal validation.

**Table 5:** AUC differences on subset Fuji_Inst1_TEST (internal validation).

	Model-F1A_F2	Model-F1A’_ F2’_GE
Model-F1A_F1B	0.029 (− 0.008, 0.068)	0.080 (0.024, 0.141)*
Model-F1A_F2		0.052 (0.008, 0.101)*

95% confidence intervals for bootstrapping differences are reported in parentheses.

*Statistical significant difference (*p*-value < 0.05).

**Figure 3:**
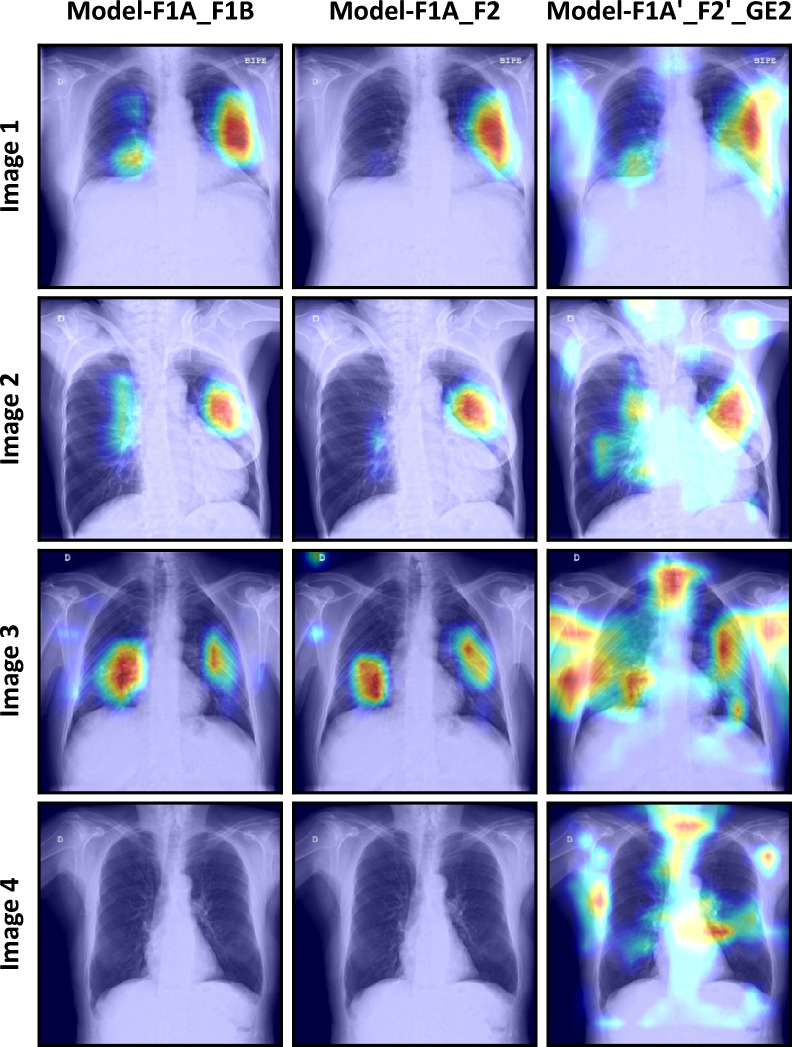
Comparison of four Grad-CAM heatmaps based on the predictions of the three models.

Grad-CAM heatmaps showed similar activation patterns between the two models. Heatmaps depicted activations over COVID-19 lung opacities, and absence of activations within any region of the image in Control patients (Fig. 3). This result suggests that both models were able to learn the radiological findings of COVID-19 and, thus, made predictions based on causal relationships.

In contrast, internal validation performances of Model-F1A_F1B and Model-F1A_F2 were 8% (*p* < 0.05) and 5.2% (*p* < 0.05) significantly higher than Model-F1A’_ F2’_GE2 internal validation performance (Table 6). Hence, the addition of images acquired by another X-ray device of a different manufacturer to the training sample decreased the algorithm’s internal validation performance.

**Table 6:** Generalization of Model-F1A_F1B across institutions and devices from different manufacturers.

Comparison	AUC difference	Shifting factor
Fuji_Inst1_ TEST vs Fuji_Inst2_TEST	0.098 (0.045, 0.154)*	Institution
Fuji_Inst2_TEST vs Care_Inst2_TEST	0.091 (0.042, 0.135)*	Image processing
GE_Inst2_TEST vs Care_Inst2_TEST	− 0.146 (− 0.171, − 0.111)*	Response function

Note that Fujifilm and Carestream devices had the same type of response function but different image processing, whereas the GE device had a different type of response function and different image processing.

*Statistical significant difference (*p*-value < 0.05).

This was also evidenced in Grad-CAM heatmaps, where Model-F1A’_ F2’_GE2 showed activation areas located outside the lungs without any clinical or radiological interpretation. Unlike Model-F1A_F1B and Model-F1A_F2, Model-F1A’_ F2’_GE2 learnt spurious relationships (confounding factors) instead of causal relationships (Fig. 3).

#### Experiment 2: evaluation of generalization

**Figure 4:**
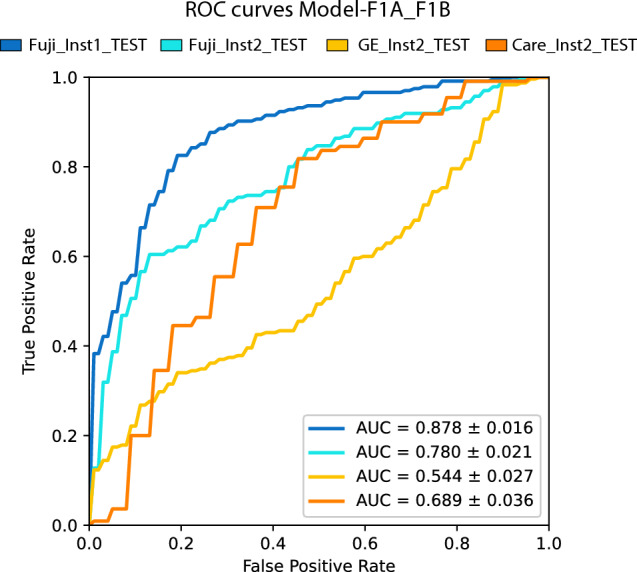
ROC curves of Model-F1A_F1B on the tests subsets: Fuji_Inst1_TEST (internal validation); Fuji_Inst2_TEST (generalization to an external institution with same X-ray device); Care_Inst2_TEST (generalization to an external institution with a X-ray device which has a different image processing but the same type of response function); and GE_Inst2_TEST (generalization to an external institution with a X-ray device which has a different image processing and a different type of response function).

Model-F1A_F1B generalized to Fujifilm and Carestream images from institution 2 (Fuji_Inst2_TEST and Care_Inst2_TEST) with a performance decrease of 9.8% (*p* < 0.05) and 18.9% (*p* < 0.05), respectively. In contrast, this model did not generalize to GE images from institution 2 (GE_Inst2_TEST), as it showed a loss in the AUC of 33.5% (*p* < 0.05) which caused the model to perform randomly (Fig. 4 and Table 6).

**Figure 5:**
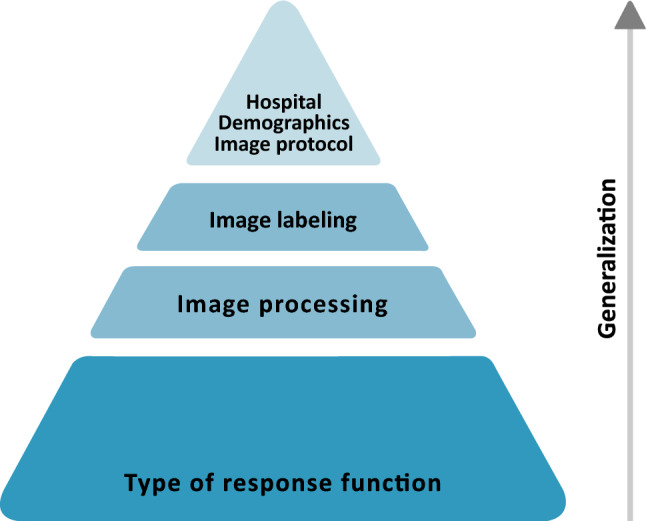
Hierarchy of factors that affect the generalization of a deep learning network in medical image classification.

Thus, Model-F1A_F1B generalized across institutions and across X-ray devices from different manufacturers with the same type of response function, however, it did not generalize across X-ray devices with different types of response function. A hierarchy of factors influencing the generalization capability of the DL network is presented in Fig. 5.

#### Experiment 3: evaluation of the dependence of CNN-features on textures

**Figure 6:**
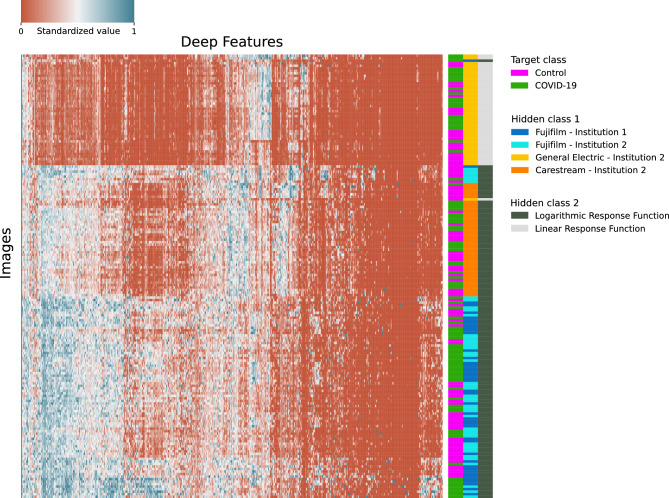
Hierarchical clustering of test subset images (Fuji_Inst1_TEST, Fuji_Inst2_TEST, GE_Inst2_TEST, Care_Inst2_TEST) based on features extracted by Model-F1A_F1B. Hierarchical clustering was generated using python's scientific graphics library, seaborn (version 0.11.1)^21^.

The hierarchical clustering algorithm grouped images from the test subsets into three evident clusters, which corresponded to images from each of the three X-ray devices used to acquire the images (Fujifilm, GE and Carestream). Radiographies acquired by both Fujifilm devices (subsets Fuji_Inst1_TEST and Fuji_Inst2_TEST) were mixed, despite being acquired in different institutions. Ultimately, images from different target classes (COVID-19 and control) were not separated (Fig. 6).

Besides, the two clusters corresponding to images acquired by the two X-ray devices with the same type of response function (Fujifilm and Carestream) were next to each other, grouped together in a higher cluster level, and separated from the cluster containing GE images, which had a different type of response function. Same results were observed when the experiment was repeated using a cropped version of the images.

In summary, the hierarchical clustering algorithm found that the feature values extracted by the pretrained ImageNet CNN were more dissimilar regarding the hidden classes (X-ray device and type of response function) than the real target classes (COVID-19 and control).

## Discussion

### Experiment 1: evaluation of internal validation performance

The similarity in performance between Model-F1A_F1B and Model-F1A_F2 suggests that institutional related factors may not have a significant impact on the internal validation performance of the algorithm. Besides, the addition of images acquired by a different model of X-ray device to the training set led to a significant performance reduction in the internal validation of Model-F1A’_ F2’_GE2. This result indicates a potentially important influence of device related factors on the algorithm’s internal validation performance.

Grad-CAM heatmaps were in line with the aforementioned results. Heatmaps of Model-F1A_F1B and Model-F1A_F2 showed activations over lung opacities in COVID-19 images and absence of activations in control images. These activation patterns suggest that those two models were able to learn causal relationships. Conversely, Model-F1A’_ F2’_GE2 did not show human-recognizable activations. COVID-19 lung consolidations were not properly identified and several activations without clinical meaning appeared both in COVID-19 and control images. In summary, Grad-CAM heatmaps also provided evidence of a variable level of influence of device related factors on the internal validation performance of the algorithm.

### Experiment 2: evaluation of generalization

This study found that a DL network can generalize across institutions and X-ray devices with the same type of response function, however, it may suffer a variable decrease in performance when deployed on external datasets. In contrast, generalization across X-ray devices with a different type of response function was not observed in this research.

Generalization of DL networks for CXRs classification to external datasets has been argued by a few authors. Pooch et al.^9^ concluded that state-of-the-art DL algorithms do not generalize to external data which differs from the data used for training. Similar to this, Zech et al.^12^ and Sathitratanacheewin et al.^10^ defend that CNNs do not generalize to external sites. Additionally, Zech et al.^12^ and Maguolo and Nanni^7^ warn that neural networks can often distinguish the dataset or the hospital where the images come from. For Maguolo and Nanni^7^, this issue is very important since most papers obtain images of each class to predict from different datasets. Trying to understand how CNNs distinguish the source of the dataset, Cohen et al.^24^ proposed discrepancies in image labeling criteria among medical centers to be the potential cause. Furthermore, for Rajpurkar et al.^14^ and Pan et al.^8^ DL algorithms for CXR classification can generalize to datasets from external institutions with a decrease in their performance. Our results agree with these last two authors.

In an attempt to shed light on the controversy surrounding the generalization of DL networks, we separately assessed the influence of multiple factors on generalization. Our research found that the X-ray device’s response function is probably the most important factor to enable generalization, followed by the device’s image processing, which hindered but did not impede the algorithm to generalize.

Furthermore, institutional related factors were also found to reduce algorithm’s performance, but to a minor extent than X-ray device related factors (Fig. 5).

### Experiment 3: evaluation of the dependence of CNN-features on textures

Hierarchical clustering showed that feature values extracted by a CNN could be highly dependent on the X-ray device that acquires the image. The reason is that each X-ray device model applies a unique image processing and has a distinct response function, which generates different textures in radiographic images. These textural differences may lead to disparities in CNN-feature values among images from different devices and vendors that could hinder generalization. Therefore, the application of DL networks to images acquired by devices from manufacturers that are different from those used to acquire the training images should be carefully accomplished.

In fact, the results of experiment 3 also suggest that the influence of X-ray devices on CNN-feature values could be even higher than the influence of the target classes or the institution. Nevertheless, the impact of this issue may be more significant in challenging classification tasks, while in relatively easy tasks, such as body parts classification in radiography, it may not pose a significant obstacle.

The dependence of CNN-feature values on the X-ray device indicates that at least some of the pre-trained CNNs extract features mainly based on textures rather than shapes. This is an important issue for generalization since shape-based features are potentially more robust and invariant than texture-based features. Accordingly, outside the medical field, Geirhos et al.^25^ previously argued that ImageNet-trained CNNs are biased towards recognizing textures rather than shapes. These authors also suggest that shape biased networks are inherently more robust than texture biased networks^25^.

Finally, this research introduces hierarchical clustering as a potentially useful tool to detect hidden classes in a dataset which can be more relevant than target classes. Therefore, training a different algorithm for each hidden class to classify the target classes instead of training a single algorithm may be a prudent approach to consider.

Taking all these findings into account, this paper argues that generalization across institutions is possible; however, the influence of the X-ray device on the performance of DL networks is highly significant. In light of these findings, we propose a new strategy for developing algorithms for interpreting radiological images: training a different algorithm for each device model. We believe that this strategy could achieve higher-performing DL models, using a smaller training dataset. This strategy could also help the algorithms to learn causal relationships, as in our research Grad-CAM heatmaps showed. In cases where acquisition equipment is unknown, hierarchical clustering can help to separate images into homogeneous clusters. This new strategy should be studied in future papers.

## Conclusion

The performance of DL algorithms in medical imaging can be influenced by mainly two types of factors: institutional and device related. On the one hand, institutional related factors are those that do not modify pixel values (labeling criteria, radiology workflow, etc.). Although these factors do not impede generalization, they can produce a relevant performance decrease when adopted in an external institution.

On the other hand, device related factors (device’s image processing, response function, and acquisition protocol) modify image pixel values, and they can have a significant impact on internal validation and generalization performances. The device’s type of response function was found to be the most critical factor, as a change on it prevented the algorithm from generalizing, while other device related factors hindered, but did not impede, generalization.

Thereby, radiography devices apply a unique image processing and response function which generate different textures in radiographic images. Hence, feature values extracted by CNNs were found to be highly dependent on the X-ray device from which the image was acquired (hidden class). This is an especially relevant issue, which may compromise generalization to external X-ray models. Clustering algorithms are deemed useful to identify hidden classes in the dataset, and we propose them as a potential strategy to evaluate CNN feature values.

### Supplementary Information

Supplementary Figures.

Supplementary Information.

## Data Availability

Data from this study were anonymized and can be made available upon reasonable request by contacting the first author at pablomenendezfernandezmiranda@gmail.com or the corresponding author.

## References

[1] Borghesi A, Roberto M (2020). Covid-19 outbreak in Italy: Experimental chest x-ray scoring system for quantifying and monitoring disease progression. Radiol. Med..

[2] Al Aseri Z (2009). Accuracy of chest radiograph interpretation by emergency physicians. Emerg. Radiol..

[3] Hwang EJ (2019). Deep learning for chest radiograph diagnosis in the emergency department. Radiology.

[4] Irvin J et al. CheXpert: A Large Chest Radiograph Dataset with Uncertainty Labels and Expert Comparison. Proceedings of the AAAI Conference on Artificial Intelligence. 33, 590–597. https://stanfordmlgroup.github.io/competitions/chexpert/. (2019). Accessed 12 March 2022.

[5] Johnson AEW (2019). MIMIC-CXR, a de-identified publicly available database of chest radiographs with free-text reports. Sci. Data.

[6] Wang L, Lin ZQ, Wong A (2020). COVID-Net: A tailored deep convolutional neural network design for detection of COVID-19 cases from chest X-ray images. Sci. Rep..

[7] Maguolo G, Nanni L (2021). A critic evaluation of methods for covid-19 automatic detection from x-ray images. Inf. Fusion.

[8] Pan I, Agarwal S, Merck D (2019). Generalizable inter-institutional classification of abnormal chest radiographs using efficient convolutional neural networks. J. Digit. Imaging.

[9] Pooch EHP, Ballester P, Barros RC, Petersen J (2020). Can we trust deep learning based diagnosis? The impact of domain shift in chest radiograph classification. Thoracic Image Analysis. TIA 2020. Lecture Notes in Computer Science.

[10] Sathitratanacheewin S, Sunanta P, Pongpirul K (2020). Deep learning for automated classification of tuberculosis-related chest x-ray: Dataset distribution shift limits diagnostic performance generalizability. Heliyon.

[11] Subbaswamy, A. & Saria, S. Counterfactual normalization: Proactively addressing dataset shift using causal mechanisms. In *34th Conference on Uncertainty in Artificial Intelligence 2018*, Vol. 2 (eds Silva, R. *et al.*) 947–957 (Association For Uncertainty in Artificial Intelligence (AUAI), 2018).

[12] Zech JR (2018). Variable generalization performance of a deep learning model to detect pneumonia in chest radiographs: A cross-sectional study. PLoS Med..

[13] Eche T, Schwartz LH, Mokrane FZ, Dercle L (2021). Toward generalizability in the deployment of artificial intelligence in radiology: Role of computation stress testing to overcome underspecification. Radiol. Artif. Intell..

[14] Rajpurkar P et al. CheXpedition: Investigating Generalization Challenges for Translation of Chest X-Ray Algorithms to the Clinical Setting. https://arxiv.org/abs/2002.11379. (2020). Accessed 12 December 2022.

[15] World Medical Association (2013). World medical association declaration of Helsinki: Ethical principles for medical research involving human subjects. JAMA.

[16] Lanca L, Silva A (2013). Digital Imaging Systems for Plain Radiography.

[17] KCARE Reports. Technical Report 05078: Quantitative evaluation of digital detectors for general radiography. https://kcare.co.uk. (2005). Accessed 8 March 2022.

[18] Calì C, Longobardi M (2015). Some mathematical properties of the ROC curve and their applications. Ricerche Mat..

[19] Selvaraju RR et al. Grad-cam: Visual explanations from deep networks via gradient-based localization. Proceedings of the IEEE International Conference on Computer Vision (ICCV). https://ieeexplore.ieee.org/document/8237336. (2017). Accessed 22 June 2022.

[20] Guess MJ, Wilson S (2002). Introduction to hierarchical clustering. J. Clin. Neurophysiol..

[21] Waskom ML (2021). seaborn: Statistical data visualization. J. Open Source Softw..

[22] LeDell E, Petersen M, van der Laan M. cvAUC: Cross-Validated Area Under the ROC Curve Confidence Intervals. R package. http://CRAN.R-project.org/package=cvAUC. (2014). Accessed 14 December 2022. 10.1214/15-EJS1035 PMC4533123 26279737

[23] Efron B, Tibshirani R (1986). Bootstrap methods for standard errors, confidence intervals, and other measures of statistical accuracy. Stat. Sci..

[24] Cohen JP, Hashir M, Brooks R, Bertrand H. On the limits of cross-domain generalization in automated x-ray prediction. Proceedings of the Third Conference on Medical Imaging with Deep Learning (PMLR). 121, 136–155. https://proceedings.mlr.press/v121/cohen20a. (2020). Accessed 05 December 2022.

[25] Geirhos R et al. ImageNet-trained CNNs are biased towards texture; increasing shape bias improves accuracy and robustness. https://arxiv.org/abs/2002.02497. (2020). Accessed 20 November 2022.

[26] Sitaula C, Hossain MB (2020). Attention-based vgg-16 model for covid-19 chest x-ray image classification. Appl. Intell..

[27] Mason D (2011). SU-E-T-33: Pydicom: An open source DICOM library. Med. Phys..

[28] Abadi M et al. TensorFlow: Large-scale machine learning on heterogeneous systems. Python Software. https://tensorflow.org. (2015). Accessed 01 March 2022.

[29] Chollet F Keras. Python library. https://keras.io. (2015). Accessed 01 March 2022.

[30] Virtanen P (2020). Fundamental algorithms for scientific computing in Python. Nat. Methods.

