# Supplementary material for: A retrospective study of deep learning generalization across two centers and multiple models of X-ray devices using COVID-19 chest-X rays
Source: Sci Rep. 2024 Jun 25;14:14657. doi: 10.1038/s41598-024-64941-5 (PMC11199585; doi:10.1038/s41598-024-64941-5)
Supplement: Supplementary file 1 — Supplementary Figures. [file 41598_2024_64941_MOESM1_ESM.pdf]

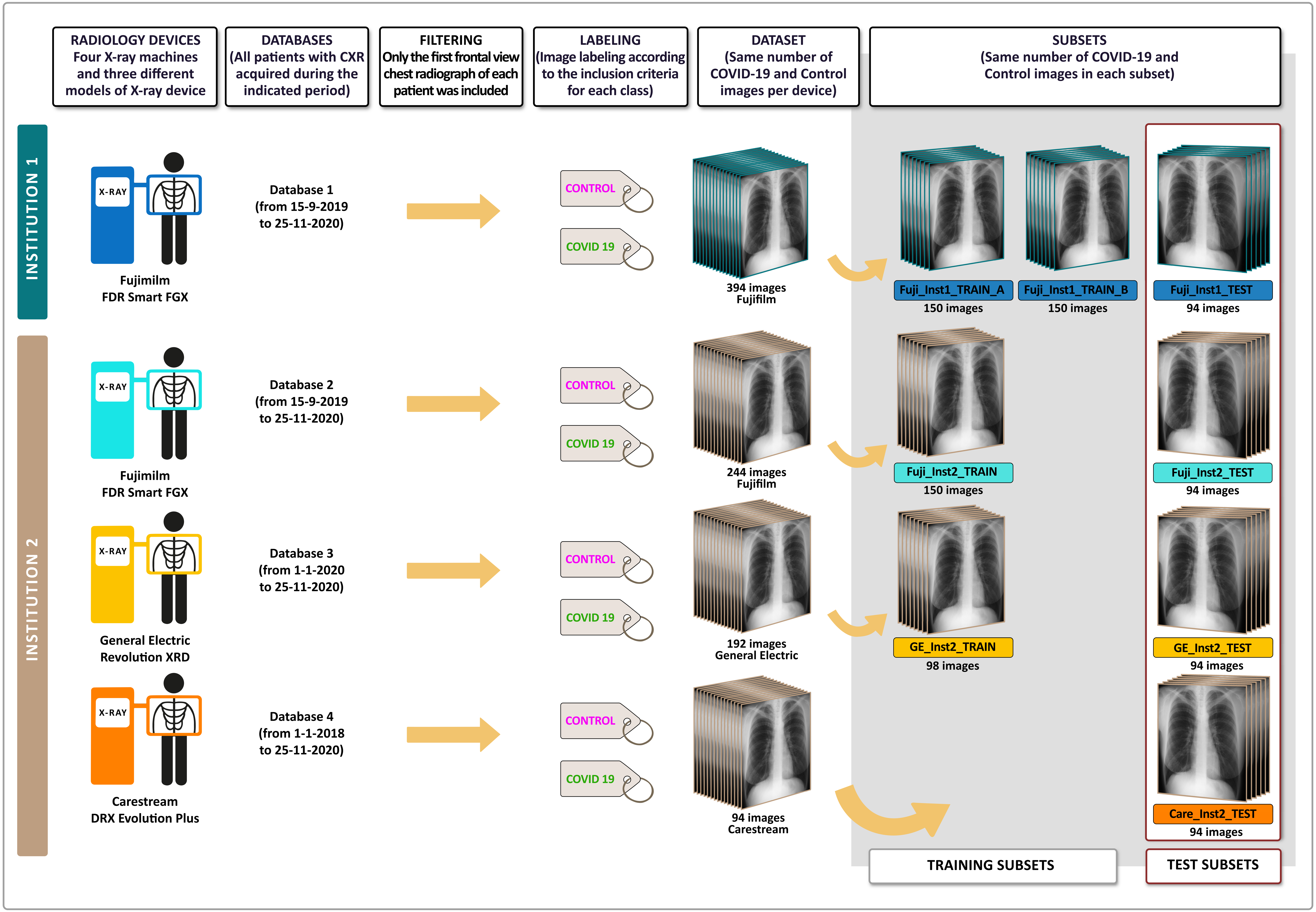

RADIOLOGY DEVICES
Four X-ray machines and three different models of X-ray device
DATABASES
(All patients with CXR acquired during the indicated period)
FILTERING
Only the first frontal view chest radiograph of each patient was included
LABELING
(Image labeling according to the inclusion criteria for each class)
DATASET
(Same number of COVID-19 and Control images per device)
SUBSETS
(Same number of COVID-19 and Control images in each subset)
INSTITUTION 1
INSTITUTION 2
X-RAY
Fujimilm
FDR Smart FGX
Database 1
(from 15-9-2019
to 25-11-2020)
CONTROL
COVID 19
394 images
Fujifilm
Fuji_Inst1_TRAIN_A
150 images
Fuji_Inst1_TRAIN_B
150 images
Fuji_Inst1_TEST
94 images
X-RAY
Fujimilm
FDR Smart FGX
Database 2
(from 15-9-2019
to 25-11-2020)
CONTROL
COVID 19
244 images
Fujifilm
Fuji_Inst2_TRAIN
150 images
Fuji_Inst2_TEST
94 images
X-RAY
General Electric
Revolution XRD
Database 3
(from 1-1-2020
to 25-11-2020)
CONTROL
COVID 19
192 images
General Electric
GE_Inst2_TRAIN
98 images
GE_Inst2_TEST
94 images
X-RAY
Carestream
DRX Evolution Plus
Database 4
(from 1-1-2018
to 25-11-2020)
CONTROL
COVID 19
94 images
Carestream
Care_Inst2_TEST
94 images
TRAINING SUBSETS
TEST SUBSETS
