# Supplementary material for: A retrospective study of deep learning generalization across two centers and multiple models of X-ray devices using COVID-19 chest-X rays
Source: Sci Rep. 2024 Jun 25;14:14657. doi: 10.1038/s41598-024-64941-5 (PMC11199585; doi:10.1038/s41598-024-64941-5)
Supplement: Supplementary file 2 — Supplementary Information. [file 41598_2024_64941_MOESM2_ESM.docx]

**Supplementary Information**

***Appendix A1. Databases and patient recruitment***

Patients were randomly selected from four databases (Figure A1). The first database contained all patients who had chest radiographs acquired by a Fujifilm FDR Smart FGX in Institution 1 between September 15^th^, 2019 and November 25^th^, 2020. The second database incorporated all patients with CXRs acquired by a Fujifilm FDR Smart FGX in Institution 2, during the same period. The third database included all patients with CXRs acquired by a GE Revolution XRD in Institution 2 between January 1^st^, 2020 and November 25^th^, 2020. The fourth database contained all patients with CXR acquired by a Carestream DRX Evolution Plus in Institution 2, from January 1^st^, 2018 to November 25^th^, 2020. Both Fujifilm and Carestream devices had a logarithmic response function, but different image processing. The GE device had a linear response function. Only the first frontal view radiograph from each patient was included to avoid potential biases that could derive from incorporating multiple images per patient.

---------------------------------------------------- Figure A1 -------------------------------------------------

**Figure A1:** Dataset collection and partition into training and test subsets.

***Appendix A2. Deep learning algorithm architecture***

CNNs with weights pretrained on ImageNet were fine-tuned with our training subsets three times. The chosen architecture was VGG16, because it showed satisfactory performance classifying COVID-19 images in previous studies [26]. On top of the VGG16, a classifier including a Global Max Pooling layer and an output single neuron with a sigmoid activation function were added. Layers were not frozen during training. The training technique was a stratified 5-fold cross-validation with the same hyperparameters during the three trainings (Figure 2). Their performance was evaluated through the AUC on the four test subsets: Fuji_Inst1_TEST, Fuji_Inst2_TEST, GE_Inst2_TEST, Care_Inst2_TEST.

***Appendix A3. Programming Resources***

Computer languages used for this research were Python 3.6.8 and R 4.1.0. Python main libraries used were Pydicom 2.1.1 [27], TensorFlow 2.0.0 [28], Keras 2.2.4 [29], and SciPy 1.5.4 [30]. The most relevant R package used was cvAUC 1.1.0 [22].
